# CON-COUR study: Interferential therapy in the treatment of chronic constipation in adults: study protocol for a randomized controlled trial

**DOI:** 10.1186/s13063-015-0752-8

**Published:** 2015-05-28

**Authors:** Véronique Vitton, Alban Benezech, Stéphane Honoré, Patrick Sudour, Nathalie Lesavre, Pascal Auquier, Karine Baumstarck

**Affiliations:** Service de Gastroentérologie, Hôpital Nord, Assistance Publique-Hôpitaux de Marseille, Marseille, France; Plateforme d’Interface Clinique, CRN2M, UMR 7286, Aix Marseille Université, Marseille, France; Unité d’expertise pharmaceutique et recherche biomédicale, AP-HM, Marseille, France; Direction de la Recherche, AP-HM, Marseille, France; Centre d’Investigation Clinique 1409, AP-HM, Aix-Marseille Université, Marseille, France; EA3279 Self-perceived Health Assessment Research Unit and Department of Public Health, AP-HM, Aix-Marseille University, Méditerranée, 27 bd Jean Moulin, Marseille, Cedex 05 F-13385 France

**Keywords:** Interferential therapy, Medical-refractory chronic constipation, Adults, Randomized controlled trial

## Abstract

**Background:**

The prevalence of chronic constipation is about 15 % in Western countries with a significant impact on quality of life and health care costs. The first-line therapy, based on medical treatment combined with laxatives and dietary rules, is often disappointing. Interferential therapy is a new treatment that has demonstrated its efficiency in the treatment of chronic constipation in children and encouraging results in adults. The primary objective of this study is to assess the efficacy of interferential therapy during 8 weeks in adult patients. The secondary objectives are to assess this new and noninvasive therapy in terms of persistence of the clinical efficacy, colonic transit time, ano-rectal manometry, patient satisfaction and quality of life (QoL), and tolerance.

**Methods/Design:**

Design: multicenter, prospective, randomized, placebo-controlled, double blind, two-parallel groups study. Setting: nine French adult gastroenterology centers. Inclusion criteria: adult patients with a history of chronic constipation refractory to medical treatment for at least 3 months. Treatment groups: (1) interferential-experimental group (effective stimulation); (2) placebo-control group (sham stimulation). Randomization: 1:1 allocation ratio. Evaluation times: inclusion (T0, randomization), baseline assessment (T1), start of stimulation (T2), intermediary assessment (T3, 4 weeks), end of stimulation (T4, 8 weeks), follow-up (T5 and T6, 1- and 6-month). Endpoints: (1) primary: short-term efficacy at T4 (treatment response defined as three or more spontaneous, complete bowel movements per week); (2) secondary: efficacy at T5 and T6, symptoms (Patient Assessment of Constipation Symptoms questionnaire), colonic transit time, anorectal manometry, patient satisfaction (analogical visual scale), patient QoL (Patient Assessment of Constipation Quality of Life Questionnaire), side/unexpected effects. Sample size: 200 individuals to obtain 80 % power to detect a 20 % difference in treatment response at T4 between the two groups (15 % of lost to follow-up patients expected).

**Discussion:**

The randomized, double-blind, placebo-controlled design is the most appropriate to demonstrate the efficacy of a new experimental therapeutic (Evidence-Based Medicine Working Group classification). National and international recommendations could be updated based on the findings of this study.

**Trial registration:**

Current controlled trials NCT02381665 (registration date: February 13, 2015).

## Background

The prevalence of chronic constipation is about 15 % in Western countries with a significant impact on quality of life and health care costs [1]. Two subtypes of constipation can be identified: slow transit constipation (STC), characterized by impaired propulsion of stool and due to dysfunction of colonic smooth muscle (myopathy) or its innervation (neuropathy), or both; and evacuation disorders, characterized by difficulty or inability with stool expulsion. They include disorders of the anorectal function, such as dyssynergic defecation, as well as structural disorders, such as rectocele, descending perineum syndrome and rectal prolapse [2]. In case of failure, few treatment options are currently available. Surgery can sometimes be discussed for intractable chronic constipation. Indeed, sub-total colectomy can be proposed in case of STC but is associated with a significant morbidity. In case of pelvic floor disorders, a specific surgical treatment can be indicated. However, surgery is invasive and has a significant morbidity, and the results are inconsistent. Recently, some studies have assessed the efficacy of sacral neuromodulation in the treatment of chronic constipation with some success, but this technique is expensive and requires the surgical implantation of a medical device [3]. More recent works, including a randomized trial, have shown the efficacy of interferential current stimulation in the treatment of chronic transit constipation in children [4–6]. This treatment is used daily, at home, and uses four adhesive surface electrodes, two abdominal (placed below the costal margin) and two paraspinal (placed between T9 and L2), producing two sinusoidal currents crossing the body, 1 h per day for 1–3 months.

To date, only one open-label study has evaluated this technique in adults and has shown encouraging results in 3 months with an efficiency in 7/11 patients (63.6 %) in the number of stools, severity score of constipation and quality of life score associated with a significant improvement in the colonic transit time measured by radiomarkers [7]. These data are of particular interest since laxative treatments are often disappointing, are expensive and may have adverse events.

These observations prompted us to establish a multicenter, prospective, two-group, placebo-controlled, double-blind, randomized study with the primary objective of assessing the efficacy of interferential therapy during 8 weeks in adult patients with severe chronic constipation. The secondary objectives are to assess this new noninvasive therapy in terms of persistence of the efficacy, colonic transit time, ano-rectal manometry, patient satisfaction and quality of life (QoL), and tolerance.

## Methods/Design

### Design

This multicenter, prospective, randomized, placebo-controlled, double-blind, two-parallel group study is performed to assess the efficacy of the use of interferential therapy: the experimental group (effective stimulation) and control group (sham stimulation). The study protocol was designed using the recommendations of the Consolidated Standards of Reporting Trials (CONSORT) statement.

### Partners

The sponsor of the study is the Assistance Publique-Hôpitaux de Marseille (AP-HM, France). The recruiting will be performed in nine French adult gastroenterology departments. The methodological support will be provided by the Clinical Research Unit (Unité Aide Méthodologique à la Recherche Clinique, AP-HM, France), the Clinical Investigation Unit (Centre d’Investigation Clinique, AP-HM, France) and the Self-perceived Health Assessment Research Unit (Aix-Marseille University, Marseille, France). The central pharmacy of AP-HM is in charge of the assignment, allocation and delivery of the devices. This work is supported by institutional grants from the French 2013 National Program of Clinical Research (Programme Hospitalier de Recherche Clinique National). All the details are provided in Table 1.

**Table 1:** French partners

Gastrointestinal specialists	Center/department
Pr. Véronique Vitton	Coordinating investigator. Public Academic Teaching Hospital Nord, Marseille
Dr. Alban Benezech	Associated investigator. Public Academic Teaching Hospital Nord, Marseille
Drs. Benoit Coffin and Jean-Marc Sabate	Public Academic Teaching Hospital Louis Mourrier, Paris
Dr. Henri Damon and Pr. François Mion	Public Academic Teaching Hospital Hospices Civils, Lyon
Pr. Michel Dapoigny	Public Academic Teaching Hospital, Clermont-Ferrand
Pr. Anne-Marie Leroi and Dr. Guillaume Gourcerol	Public Academic Teaching Hospital, Rouen
Pr. Thierry Piche	Public Academic Teaching Hospital, Nice
Dr. Michel Queralto	Clinique des Cèdres, Cornebarrieu
Pr. Laurent Siproudhis	Public Academic Teaching Hospital, Rennes
Pr. Frank Zerbib	Public Academic Teaching Hospital, Bordeaux
Multidisciplinary team	
Pr. Pascal Auquier	Public Health, Public Academic Teaching Hospital, Marseille
Dr. Karine Baumstarck	Clinical Research Unit, Public Academic Teaching Hospital, Marseille
Dr. Nathalie Lesavre	Clinical Investigation Center, Public Academic Teaching Hospital, Marseille
Drs. Stéphane Honoré and Anita Cohen	Pôle Pharmacie, Public Academic Teaching Hospital, Marseille

### Participants

The details of the inclusion and exclusion criteria are provided in ‘List of selection criteria’. The main inclusion criteria are adult patients with a history of medical therapy-refractory chronic constipation for more than 6 months, defined as two or fewer spontaneous, complete bowel movements per week for a minimum of 6 months or as a sensation of incomplete evacuation or straining during defecation. The main exclusion criteria are patients with constipation secondary to anorectal malformations, to colorectal or anal organic lesions or to a pelvic floor disorder, to a drug, to neurologic, endocrine or metabolic disorders.

**List of selection criteria**

Inclusion criteria

- Adults patients (≥18 years of age) of either sex
- Subjects with a history of chronic constipation defined as: two or fewer spontaneous, complete bowel movements per week for a minimum of 6 months before the screening visit or as a sensation of incomplete evacuation or straining during defecation with at least 25 % bowel movements
- Subjects with chronic constipation lasting for more than 6 months
- Subjects with chronic constipation refractory to medical treatment for at least 3 months (failure or intolerance of medical treatment)
- Subjects have to interrupt any laxative treatment but during the study, patients are allowed, in case of absence of bowel movements for 3 or more consecutive days, to take up 15 mg of bisacodyl (Dulcolax, Boerhinger Ingelheim) as rescue medication
- Subjects affiliated to or beneficiary of a social security system
- Subjects who have signed written informed consent

Exclusion criteria

- Minors or pregnant or breast-feeding women
- Subjects with chronic constipation secondary to anorectal malformations, to colorectal or anal organic lesions or to a pelvic floor disorder considered by the investigator as necessitating a surgical treatment (rectal prolapse exteriorized, rectocele, enterocele)
- Subjects with current implanted cardiac pacemakers, defibrillators, cardiac pumps, spinal stimulators or other implanted electronic devices
- Subjects with chronic constipation secondary to a drug, to neurologic, endocrine or metabolic disorders
- Subjects with a history of partial colectomy
- Subjects with megacolon, megarectum, colonic inertia
- Subjects with skin lesions preventing the installation of the electrodes
- Women without effective contraception (hormonal or intrauterine device)
- Subjects misunderstanding the written and spoken French language
- Subjects participating in another biomedical research protocol

### Treatments

The device procedure (effective or sham) will be blinded; neither the patient nor the physician responsible for the prescription will be informed of the nature of the device. Whatever the group (control or experimental), the patients will follow the same procedure, will receive a device looking exactly the same and will have the same follow-up. This design has already been used in randomized studies using electrical stimulation [4, 8]. The practitioner that will give the instructions concerning the use of the device will be aware of the sham or effective stimulation since he/she will need to give different instructions according to the device (sham or effective). The feasibility of this treatment has previously been demonstrated [7].

#### Interferential-experimental group: effective stimulation

The patients will receive stimulation 1 h per day every day at home during 8 weeks. There is no “dose adjustment;” if the stimulation is not well tolerated, it will be stopped according to the opinion of the practitioner. The stopping criteria are as follows: severe pain or neurological symptoms (paresthesia, burning, shaking). Unblinding of treatment could be done by contacting the coordinating investigator.

#### Placebo-control group: sham stimulation

The patients will receive the sham device without any stimulation 1 h per day every day at home for 8 weeks. The device will be the same as the one used in the experimental group but there will not be any stimulation. The device will not be active in this group; thus, no side effects or intolerance is expected. However, if any side effects are reported (e.g., pain), the stimulation will be stopped in accordance with the cessation criteria detailed below. Unblinding of treatment could be made as described above.

#### Concomitant permitted and prohibited medications

In case of absence of bowel movements for 3 or more consecutive days, the patients are allowed to take up 15 mg of Bisacodyl (Dulcolax, Boerhinger Ingelheim) as rescue medication because it is simple to use and has satisfactory tolerance and rapid action [9]. All other laxative drugs are forbidden during the study.

#### Medical device

The interferential therapy device uses a 6-V battery-operated interferential stimulating machine (Flexitim IF Tenscare, Fuji Dynamics, Hong Kong, CE mark 0473, Market authorization December 2009). The device is marketed in France by A-Legrand Co. (http://www.a-legrand.com). Interferential treatment delivers a 4-kHz carrier frequency, a beat frequency of 80–160 Hz and an intensity of less than 33 mA. The stimulation will be done according to the procedure described by Ismail et al. [6]. This technique uses four adhesive surface electrodes, two abdominal (placed below the costal margin) and two paraspinal (placed between T9 and L2), producing two sinusoidal currents crossing the body. To ensure the proper placement of the electrodes, the first stimulation must be made in the presence of the practitioner. During the 8-week treatment period, the stimulation protocol is home-based and self-applied by the patient for 1 h per day every day.

### Recruitment and follow-up

#### Screening and inclusion

During a 2-week observational period, the eligible patients will be asked to report the number of spontaneous, complete bowel movements per week to the physicians (partners of the study) in a bowel diary (T0). The patients who meet all the inclusion and exclusion criteria will be randomized into one of the two treatment groups after completing the consent form and getting it to the investigator.

#### Randomization

Computer-generated randomized lists will be drawn up before the beginning of the study, using a permuted block design, under the responsibility of the clinical research unit (AP-HM). The randomization will be stratified by center (1:1 allocation ratio). The allocation sequences will be sequentially numbered. The pharmacist responsible for the medical device distribution in the participating centers will take care of the lists. Prior to the study period, the participant, the treating medical staff and the investigators will all be unaware of the allocation.

#### Follow-up and data collection

The evaluation will be performed at six different time points: baseline (T1), initial visit (T2, beginning of the stimulation), intermediary visit (T3, 4 weeks after T2), end of stimulation (T4, 8 weeks after T2), and 1 and 6 months after the end of stimulation (T5 and T6). Minimal data of lost to follow-up patients will be collected to determine whether they differ from the others. The study procedure and data collection are detailed in Table 2.

**Table 2:** Study procedure

	T0	T1	T2	T3	T4	T5 and T6
Consent	x					
Randomization	x					
Clinical examination	x	x	x	x	x	x
Colonic transit		x			x	
Ano-rectal manometry		x			x	
Stimulation						
Constipation symptoms (diary, PAC-SYM)		x	x	x	x	x
Quality of life (PAC-QOL)		x	x	x	x	x

*T0* pre-inclusion, *T1* baseline assessment (T0 ± 15 days), *T2* beginning of stimulation (T1 ± 15 days), *T3* intermediary assessment (T0 ± 4 weeks), *T4* end of stimulation (T0 ± 8 weeks), *T5* follow-up at 1 month, *T6* follow-up at 6 months and end of study

### Endpoints/Evaluation criteria

#### Primary endpoint

The primary endpoint is the short-term efficacy (response to treatment) after 8 weeks of stimulation. A subject will be classified as a responder when he/she has three or more spontaneous, complete bowel movements per week during the 8 weeks of the trial. The primary endpoint was chosen considering the last randomized trial on severe chronic constipation published in the New England Journal of Medicine [9].

#### Secondary endpoints

Efficacy will be assessed by the following endpoints:

- Long-term efficacy (response to treatment) at 1 (T5) and 6 months (T6).
- Constipation symptoms will be assessed using the Patient Assessment of Constipation Symptoms questionnaire (PAC-SYM) [10]. This questionnaire includes 12 constipation-related symptoms scored on three subscales: stool, abdominal or rectal symptoms. For the overall scale and for each subscale, scores can range from 0 (symptoms absent) through 4 (very severe symptoms).
- Colonic transit time will be measured according to the Bouchoucha technique [11]. With this technique, the subjects ingest ten markers for 6 days at regular times. A plain film of the abdomen is performed at the 7th day. The number of persistent markers is analyzed in each area of interest (colonic segment). The total and segmental transit time is calculated by multiplying the number of markers by segment by 2.4. A slow transit time is defined by a total transit time greater than 91.2 h for women and 76.8 h in men.
- Ano-rectal manometry will be realized according to each center’s usual procedure. The following parameters will be collected: resting pressure and increment in the voluntary contraction measured in mmHg, maximal rectal tolerable volume in ml and presence of an anismus.
- Self-perception of the subject will be assessed using a patient global assessment of efficacy of the treatment based on an analogical visual scale (AVS) from 0 (not satisfied at all) to 5 (completely satisfied).
- The patients’ quality of life will be assessed using the Patient Assessment of Constipation Quality of Life (PAC-QOL) questionnaire [12]. This self-reported questionnaire includes 28 items related to the effects of constipation on the daily life on four subscales: physical discomfort, psychosocial discomfort, worries and concerns, and satisfaction. For each item, scores can range from 0 through 4 with lower scores indicating a better quality of life.
- Tolerance will be analyzed from the side effects or unexpected effect such as pain (local, ventral or dorsal) or neurological symptom such as paresthesia, burning and shaking.

### Pharmaceutical aspects

Devices (both effective or sham) and electrodes will be labeled and numbered according to the randomization list and sent to the dispensing pharmacist of each investigating center under the responsibility of the Hospital Pharmacy of AP-HM. At the end of the study, devices will be returned to the hospital coordinating pharmacy of the sponsor.

### Statistical considerations

#### Sample size, power and statistical methods

The sample size was determined to obtain an 80 % power to detect a 20 % difference in treatment response at 8 weeks between the two groups (15 % in the control group). This difference based on previous reports [1, 3, 9] has been considered to be clinically significant. With the threshold for statistical significance set at a *p*-value of 0.05, assuming that a potential 15 % of patients will be lost to follow-up, these calculations showed that 190 patients are needed (95 per group). A total of 200 individuals will be included.

#### Data analysis

The data will be analyzed using SPSS version 17.0 software. The patients found to be eligible but not included in the study will be described and compared with the included patients. The patients who present at least one of the following conditions will not be included in the final analysis: patients inappropriately included despite providing consent and patients who withdrew their consent. The full analysis population (including all subjects who will be randomized and will be at least evaluated at T2) will be used in the primary analysis, and the *per protocol* population (including all subjects who will be randomized and will not have major protocol deviations) will be used in the secondary analysis to assess the robustness of the results. No interim analysis is planned. The normality of these parameters will be estimated using frequency histograms and the Shapiro test. The baseline parameters will be compared between the two groups (‘control’ and ‘experimental’) using the chi^2^ test or Fisher’s exact test for categorical variables and Student’s *t* test for continuous variables.

The proportion of treatment response at 8 weeks (patients with three or more spontaneous, complete bowel movements per week) will be calculated for each group and compared using the chi^2^ test or Fisher’s exact test for categorical variables. The center effect will be tested. The same procedure will be performed for the long-term efficacy. PAC-SYM scores, colonic transit times, manometric parameters, self-perception efficacy on the AVS and PAC-QOL scores will be compared between the two groups using Student’s test for continuous variables if applicable (or using nonparametric tests and the Mann-Whitney test). Changes between each initial score and the score at T3, T4, T5 and T6 will be compared between the two groups, and the analysis of variance for repeated measurements will be performed to compare the changes in the scores over time between the two groups. Multivariate analysis using logistic regression models will be performed to determine variables potentially linked to treatment response. Variables relevant to the models will be selected based on their clinical significance and/or a threshold *p*-value ≤ 0.2 in the univariate analysis. The final models will estimate the odd ratios and 95 % confidence intervals. All of the tests will be two-tailed with a 5 % significance level.

### Ethical aspects, laws and regulations

The study will be conducted in accordance with the Helsinki Declaration and the French laws and regulations (Code de la Santé Publique, article L.1121-1/Loi de Santé Publique n°2004-806 du 9 août 2004 relative à la politique de santé publique et ses décrets d’application du 27 août 2006) and the International Conference on Harmonization (ICH) E6 Guideline for Good Clinical Practice. Regulatory monitoring will be performed by the sponsor. The sponsor needed the approval of the French authorities, including the French ethics committee (Comité de Protection des Personnes Sud Méditerranée, reference number 14 89) and the French drug and device regulation agency (Agence Nationale de Sécurité du Médicament, reference number DMTCOS/DMCOSM/SV/2014-A01359-38) before beginning the study. The ClinicalTrials.gov identifier is NCT02381665. Informed consent will be obtained from all subjects.

## Discussion

To date, no randomized study has determined the efficacy and tolerance of interferential therapy, a noninvasive and nonpharmacological treatment, in adult patients suffering from chronic constipation refractory to medical treatment. If its effectiveness is demonstrated, it will provide, for the first time, a new noninvasive step for patients with laxative treatment failure before considering surgical treatment. We will use a randomized, double-blind, placebo-controlled design, which is the most appropriate design to demonstrate the efficacy of a new experimental intervention in accordance with the Levels of Evidence classification of the Evidence-Based Medicine Working Group (Oxford Centre for Evidence-Based Medicine, table of Evidence Working Group, http://www.cebm.net/index.aspx?o=5653, May 26, 2015). In the future, national and international recommendations could be updated based on our findings.

However, some issues related to the content of the protocol study should be discussed.

A parallel design has been adopted, although an intra-individual crossover design is generally considered to be the most appropriate to assess the efficacy of a new analgesic strategy because of the well-known patient variation in the subjective perception of constipation symptoms. In the crossover study, each subject acts as his/her own control. Several limitations of crossover trials have led us to avoid this design [13]. The crossover design is suitable for patients in stable condition, which is not the case for patients with chronic constipation. Moreover, we suspect that the treatment may have a remnant effect that may alter the response to subsequent treatments inducing that subjects may not be in a comparable condition at the start of each treatment period. Lastly, the crossover design does not allow the assessment of long-term efficacy.

In the current study, patients receiving the sham device will not feel any stimulation, and they will use the device during a total time of 1 h for 8 weeks. The sham device will be programmed in this way, and its external aspect will not differ from the effective device. Patients with the effective stimulation will feel the stimulation during the whole stimulation period (1 h for 8 weeks). However, since the instructions need to differ between the two patient groups, the practitioner that will give the instructions must absolutely differ from the one who will follow the patients. All patients will be told that they may or may not feel something with the stimulation because people always respond differently to the stimulation. Because only one practitioner will know which device is attributed to the patient, participants will be instructed not to reveal or discuss the type of treatment characteristics they are experiencing with the other practitioner. The other option would have been to use a sham stimulator inducing a short (few seconds or minutes) stimulation before stopping. Our choice is supported by two main reasons: The first reason is to be in accordance with the literature. Indeed, a recent review of the literature on randomized controlled trials about transcutaneous electric nerve stimulation showed that the majority of the studies reported using an inactive device looking identical in appearance to the active one [14]. The second reason is supported by the physiological effect of somato-sympathetic reflexes. Indeed, some studies have demonstrated that stimulating the skin could induce somato-visceral reflexes that can modify visceral functions. Since these activities can be induced by a very short stimulation, it seems that, to be a real control, a sham stimulator should not induce any stimulation [15–17].

Concerning the patient characteristics, the selection criteria chosen are usual criteria to evaluate a new treatment for chronic constipation defined according to the Rome criteria. However, what can be different from some other studies here is that we choose to include both slow transit constipation (STC), characterized by impaired propulsion of stool, and also evacuation disorders, characterized by difficulty or inability with stool expulsion [2]. Although the underlying mechanism is not the same, these two types of constipation are linked. Indeed, slow transit constipation may induce the production of dehydrated and small hard stools that are difficult to evacuate, thus inducing defecatory disorders. In the same manner, in case of evacuation disorders, the ano-colonic reflex is not correctly stimulated, and it can slow down the colonic transit. According to the usual imbrication of these two mechanisms, especially in clinical practice, we choose to include both of these types of chronic constipation.

The treatment procedure chosen is the same initially described by Chase et al. in children [5]. Indeed, to our knowledge, this team is the only one that has published this technique with a very interesting success rate confirmed in a randomized study [4].

The treatment response will be assessed according to both clinical and radiological criteria. The primary criterion chosen is a clinical one and will be the short-term efficacy at 8 weeks defined as the response to treatment. A subject will be classified as a responder when he/she has three or more spontaneous, complete bowel movements per week during the 8 weeks of the trial. This primary end point was chosen considering the last randomized trial on severe chronic constipation published in the New England Journal of Medicine [9]. To obtain a complete assessment of the interferential therapy effect, we complement this primary criterion with clinical and radiological efficacy endpoints. In conclusion, the results of this randomized trial are expected to confirm that interferential therapy may be a new noninvasive treatment in chronic constipation.

## Trial status

At the time of manuscript submission, the status of the trial was ‘not yet recruiting’.

## Abbreviations

ANSM: Agence Nationale de Sécurité du Médicament
AP-HM: Assistance Publique-Hôpitaux de Marseille
AVS: analog visual scale
CONSORT: Consolidated Standards of Reporting Trials
ICH: International Conference on Harmonization
PAC-QOL: Patient Assessment of Constipation Quality of Life Questionnaire
PAC-SYM: Patient Assessment of Constipation Symptoms questionnaire
RCT: Randomized controlled trial
STC: Slow transit constipation.

## References

[1] Mugie SM, Benninga MA, Di Lorenzo C (2011). Epidemiology of constipation in children and adults: a systematic review. Best Pract Res Clin Gastroenterol.

[2] Rao SS (2008). Dyssynergic defecation and biofeedback therapy. Gastroenterol Clin North Am.

[3] Kamm MA, Dudding TC, Melenhorst J, Jarrett M, Wang Z, Buntzen S (2010). Sacral nerve stimulation for intractable constipation. Gut.

[4] Clarke MC, Chase JW, Gibb S, Robertson VJ, Catto-Smith A, Hutson JM (2009). Decreased colonic transit time after transcutaneous interferential electrical stimulation in children with slow transit constipation. J Pediatr Surg.

[5] Chase J, Robertson VJ, Southwell B, Hutson J, Gibb S (2005). Pilot study using transcutaneous electrical stimulation (interferential current) to treat chronic treatment-resistant constipation and soiling in children. J Gastroenterol Hepatol.

[6] Ismail KA, Chase J, Gibb S, Clarke M, Catto-Smith AG, Robertson VJ (2009). Daily transabdominal electrical stimulation at home increased defecation in children with slow-transit constipation: a pilot study. J Pediatr Surg.

[7] Queralto M, Vitton V, Bouvier M, Abysique A, Portier G (2013). Interferential therapy: a new treatment for slow transit constipation. A pilot study in adults. Colorectal Dis.

[8] Leroi AM, Siproudhis L, Etienney I, Damon H, Zerbib F, Amarenco G (2012). Transcutaneous electrical tibial nerve stimulation in the treatment of fecal incontinence: a randomized trial (CONSORT 1a). Am J Gastroenterol.

[9] Camilleri M, Kerstens R, Rykx A, Vandeplassche L (2008). A placebo-controlled trial of prucalopride for severe chronic constipation. N Engl J Med.

[10] Frank L, Kleinman L, Farup C, Taylor L, Miner P (1999). Psychometric validation of a constipation symptom assessment questionnaire. Scand J Gastroenterol.

[11] Bouchoucha M, Berger A, Francoual G (1989). Mesure du temps de transit colique total et segmentaire à l'aide d'une seule radiographie de l'abdomen sans préparation et d'un seul type de marqueurs. Gastroenterol Clin Biol.

[12] Marquis P, De La Loge C, Dubois D, McDermott A, Chassany O (2005). Development and validation of the patient assessment of constipation quality of life questionnaire. Scand J Gastroenterol.

[13] Senn SJ (2002). Cross-over trials in clinical research.

[14] Bennett MI, Hughes N, Johnson MI (2011). Methodological quality in randomised controlled trials of transcutaneous electric nerve stimulation for pain: low fidelity may explain negative findings. Pain.

[15] Sato A, Sato Y, Sugimoto H, Tervi N (1977). Reflex changes in the urinary bladder after mechanical and thermal stimulation of the skin at various segmental levels in cats. Neuroscience.

[16] Sato A, Sato Y, Suzuki A, Uchida S (1993). Neural mechanisms of the reflex inhibition and excitation of gastric motility elicited by acupuncture-like stimulation in anesthetized rats. Neurosci Res.

[17] Sato A, Schmidt RF (1987). The modulation of visceral functions by somatic afferent activity. Jpn J Physiol.

